# Surveillance strategy of Barrett's esophagus in the Asian region with particular reference to its locoregional epidemiology

**DOI:** 10.1002/jgh3.12350

**Published:** 2020-05-12

**Authors:** Jia Di, Neel Sharma, Lady K M Pérez, Jun Zhang, Khek‐Yu Ho

**Affiliations:** ^1^ Department of Medicine, Yong Loo Lin School of Medicine National University of Singapore Singapore Singapore; ^2^ Department of Gastroenterology Second Affiliated Hospital of Xi'an Jiaotong University Xi'an China; ^3^ Department of Gastroenterology Queen Elizabeth Hospital Birmingham Birmingham UK; ^4^ Department of Internal Medicine Cleveland Clinic Cleveland Ohio USA

**Keywords:** Asian region, Barrett's esophagus, epidemiology, surveillance

## Abstract

Barrett's esophagus (BE) is a premalignant condition associated with the development of esophageal adenocarcinoma (EAC). Over the past decade, BE and its associated neoplasia has increased in prevalence globally. Current surveillance guidelines aimed to detect and treat BE‐associated dysplasia early in the hope of improving the morbidity and mortality of the condition. However, due to the lack of long‐term data and the proven benefit that surveillance actually improves mortality from EAC, the guidelines of the United States and Europe are slightly different. This review will focus on discussing the surveillance strategy for BE appropriate for the Asian region, taking into account the unique epidemiologic features of this disease in the Asian region.

## Graphical abstract

Current surveillance guidelines aim to detect Barrett's esophagus‐associated dysplasia early. However, due to the lack of long‐term data, and proven benefit that surveillance actually improves mortality from esophageal adenocarcinoma, the guidelines coming from the US and Europe are slightly different. This review will discuss the surveillance strategy for Barrett's esophagus appropriate to the Asian region, taking into account the unique epidemiologic features of this disease in the Asian region.

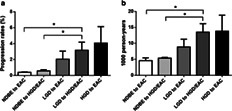

## Introduction

Barrett's esophagus (BE) is a condition in which the stratified squamous epithelium in the lower esophagus undergoes metaplastic alteration to become a columnar epithelium. Whether goblet cells are required for the diagnosis of BE has long been debated and will continue to remain so for the foreseeable future. What is generally accepted is that BE is a precursor lesion of esophageal adenocarcinoma (EAC), with the estimated risk being 0.12% per annum.^1^ Despite the low risk of progression to EAC, the incidence of EAC has risen globally. In western countries, it has risen sixfold over the past 40 years.^2^, ^3^ As the prognosis of EAC is poor, with an estimated 5‐year survival of 10–15%,^4^ early detection of dysplastic lesions, which are amenable to endoscopic therapy, remains the mainstay of the management of BE. To detect Barrett's dysplasia, a surveillance strategy for BE, which is sustainable and cost‐effective, is paramount. The strategy may vary between populations depending on the locoregional epidemiologic characteristics and risk of progression from BE to EAC. In this review, we will first describe the global epidemiology of BE and highlight the risk factors for neoplastic progression. We will then summarize and compare the surveillance strategies used internationally. Finally, we will discuss the surveillance strategy for BE appropriate for the Asian region, taking into account the unique epidemiologic features of this disease in the Asian region.

## Epidemiology of BE

The prevalence of BE has increased dramatically in recent decades, with a range estimated from 0.7 to 5.6% globally.^5^, ^6^, ^7^ The annual estimated incidence rate of BE in the general population is 1–2%, which is approximately 9.9/1000 person‐years.^7^, ^8^ The malignant transformation of BE into EAC appears to go through a series of histopathological stages that are classified as nondysplastic BE (NDBE), low‐grade dysplasia (LGD), and high‐grade dysplasia (HGD). The incidence of NDBE ranges from 0.12 to 0.25%,^9^ LGD 0.29–0.5%,^10^ HGD 0.22–1.5%,^9^, ^11^ and EAC 0.26–1.2%.^9^, ^12^

## Risk of neoplastic progression

**Figure 1:**
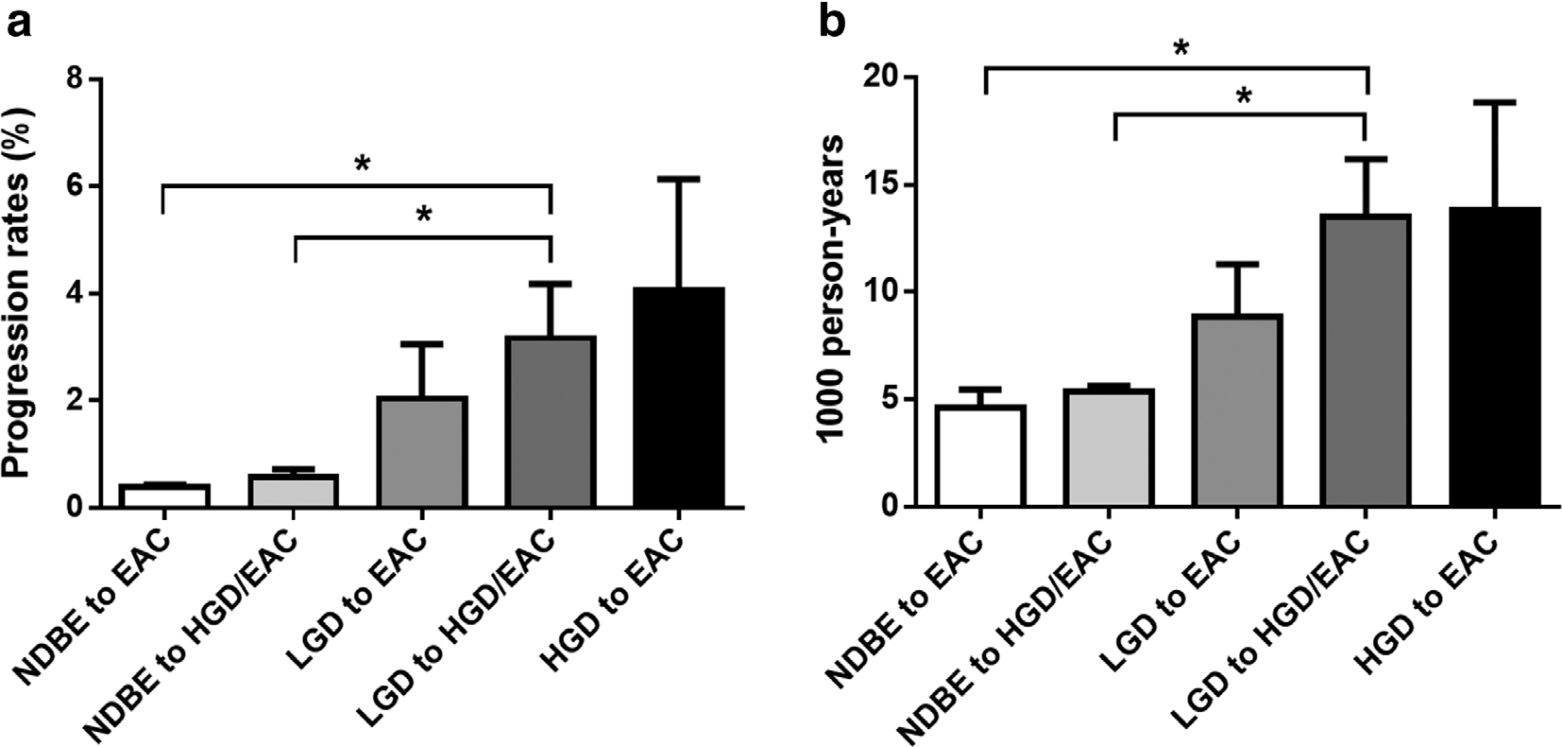
Risk of neoplastic progression in BE. (a) The progression rates of NDBE, LGD, and HGD by percentage. (b) The progression rates by 1000 person‐years. BE, Barrett's esophagus; EAC, esophageal adenocarcinoma; HGD, high‐grade dysplasia; LGD, low‐grade dysplasia; NDBE, nondysplastic Barrett's esophagus. Mean ± SE, *P<0.05, one‐way ANOVA, and the Student's *t*‐test.

Over the past 40 years, the incidence of EAC has increased more than sixfold in Western countries and continues to increase in the Asian region.^2^, ^13^ It is worth noting that the presence and grade of dysplasia increase the progression rate to EAC considerably^9^, ^12^, ^14^, ^15^, ^16^ (Fig. 1). For NDBE, the average progression rate to HGD/EAC is 0.6%, giving a risk of 5.4 per 1000 person‐years. The average progression rate from LGD to HGD/EAC is 3.2% (risk, 13.5 per 1000 person‐years), while that from HGD to EAC is 4.1% (risk, 13.8 per 1000 person‐years)^9^, ^12^, ^14^, ^15^, ^16^ (Fig. 1a,b). The risk of neoplastic progression is also influenced by other factors. It is higher in patients with coexisting intestinal metaplasia (IM), that is, columnar epithelium with concomitant goblet cells, than in those without IM. A 7‐year follow‐up study of a large cohort of BE patients noted a higher incidence of esophageal/gastric cardia cancer or HGD among patients with IM (0.38% per year) than in those without IM (0.07% per year).^17^ The risk of cancer progression is also higher in patients with long‐segment BE (LSBE, ≥3 cm) than in those with a short‐segment disease (SSBE, <3 cm). For example, a meta‐analysis showed that the annual incidence of EAC was 0.33% in LSBE *versus* 0.19% in SSBE.^18^

## Global surveillance strategies for BE

The aim of surveillance is the early detection of BE‐associated dysplasia in the hope of early treatment. The surveillance intervals based on guidelines of the United States and Europe are slightly different. For NDBE, the American College of Gastroenterology (ACG), American Society of Gastrointestinal Endoscopy (ASGE), and American Gastroenterological Association (AGA) all recommended surveillance at 3–5‐year intervals.^4^, ^19^, ^20^ In contrast, British Society of Gastroenterology (BSG), European Society of Gastrointestinal Endoscopy (ESGE), and Societe Francaise d'Endoscopie Digestive (SFED) guidelines recommend surveillance intervals that are based on the length of the BE segment.^21^, ^22^, ^23^, ^24^ The BSG guideline recommends a surveillance interval of 3–5 years for short‐segment (<3 cm) IM and 2–3 years for long‐segment (≥3 cm) IM. ESGE guideline on BE surveillance is slightly more conservative and recommends surveying SSBE every 5 years and LSBE every 3 years. For BE longer than 10 cm, ESGE advised follow‐up to be carried out at expert centers. SFED guideline is similar to that of ESGE in terms of surveillance interval for SSBE, that is, every 5 years; it differs from BSG guideline in terms of LSBE by differentiating the intervals in LSBE according to its length. The recommended surveillance interval for segment of 3–6 cm is 3 years and for segments longer than 6 cm is 2 years. LGD, BSG, ESGE, and Australia guidelines all recommend surveying the patients at a 6‐month interval.^23^ There are differences among guidelines with regard to the surveillance strategies for HGD. AGA, ASGE, ACG, and ESGE guidelines^4^, ^19^, ^20^, ^22^ recommended surveying HGD at 3 months, while the preferred strategy by BSG and Australia guidelines is endoscopic therapy^23^, ^24^ (Table 1).

**Table 1:** Surveillance guidelines for BE

Guidelines	NDBE	IND	LGD	HGD	HGD/IMC
AGA^19^	3–5 years	NA	6–12 months	3 months	Endoscopic therapy
ASGE^20^	3–5 years	NA	6 months, then every 12 months	3 months	Endoscopic therapy
ACG^4^	3–5 years	3–6 months, then every 12 months	6–12 months	3 months	Endoscopic or surgical therapy
Australia^23^	<3 cm, 3–5 years	6 months	6 months	Endoscopic therapy preferred	Endoscopic or surgical therapy
	≥3 cm, 2–3 years				
BSG^24^	<3 cm IM, 3–5 years	6 months	6 months	Endoscopic therapy preferred	Endoscopic therapy preferred
	≥3 cm IM, 2–3 years				
ESGE^22^	<1 cm, no surveillance	6 months	6 months	3 months, then every 12 months	Adjuvant or surgical therapy
	1–3 cm, 5 years				
	3–10 cm, 3 years				
	>10 cm, expert center				
SFED^21^	<3 cm, 5 years	NA	6 months, then every 12 months	Endoscopic or surgical therapy	Endoscopic or surgical therapy
	3–6 cm, 3 years				
	>6 cm, 2 years				

ACG, American College of Gastroenterology; AGA, American Gastroenterology Association; ASGE, American Society of Gastrointestinal Endoscopy; BE, Barrett's esophagus; BSG, British Society of Gastroenterology; ESGE, European Society of Gastrointestinal Endoscopy; HGD, high‐grade dysplasia; IM, intestinal metaplasia; IMC, intramucosal carcinoma; IND, indefinite for dysplasia; LGD, low‐grade dysplasia; NA, not applicable; NDBE, nondysplastic Barrett's esophagus; SFED, French Society of Digestive Endoscopy.

## Epidemiology of BE and its related neoplasia in the Asian region

In the Asian region, the prevalence of BE varies from 0.06 to 5%,^25^ which is lower compared to that of Western countries, such as those in Europe and North America.^26^ However, like the West, the prevalence of BE in the Asian region is rising. Shiota *et al*. undertook a systematic review and meta‐analysis in an East Asian setting and noted a prevalence of endoscopic BE of 7.8% across symptomatic and screening studies, with subsequent histological confirmation of 1.3%. They demonstrated an increase in prevalence; however, the majority of pathology was short segmented in nature.^27^ In support of Shiota's finding, current incidence comparisons across Asia have also shown an increase in Singapore and Japan. There has been no significant change in the incidence of BE in Taiwan and Korea in recent decades. The decline in the incidence of BE in Hong Kong is an exception.^26^ Data on the incidence rates of the transformation of BE to dysplasia or EAC in the Asian populations are limited. In a hospital‐based study from Taiwan of 51 patients with NDBE who had undergone surveillance endoscopy for a mean follow‐up of 3.7 years, the incidence rate of development of LGD is 2.9% per year and of EAC is 0.4% per year. While the study has concluded that their incidence rates are similar to those of western studies, their findings are limited by the small number of BE and LGD and EAC, which may have resulted in an overestimation.^28^

## Surveillance strategy in the Asian region

The rationale for western guidelines recommending endoscopic surveillance of NDBE at 2–5 yearly intervals has been questioned mainly because of the low absolute annual risk of BE‐associated EAC of 0.1% and the lack of clarity on whether surveillance actually improves mortality from EAC.^1^ In the East, the risk of malignant progression of BE to dysplasia or EAC is expected to be the same or lower. Furthermore, studies from the East have consistently shown a predominance of SSBE among patients with BE.^29^ These reasons, plus the lack of proven benefit in endoscopic surveillance of NDBE, lead to the Asia‐Pacific guidelines^25^ not recommending the performance of surveillance endoscopy for NDBE. However, if a decision is made that surveillance should be performed for some patients with NDBE, the Asia‐Pacific guidelines advise performing endoscopy every 3–5 years. The authors of the present review article would like to suggest that surveillance endoscopy of NDBE in the Asian region should be stratified according to the length of Barrett's segment. A 5‐year surveillance interval for nondysplastic SSBE and 3‐year interval for LSBE are a reasonable approach. The authors of the present review article agree with the Asia‐Pacific guidelines recommending the performance of endoscopic surveillance for LGD of every 6 months and the performance of endoscopic resection of ablation for HGD.

## Conclusion

Both the prevalence and incidence rates of BE have increased in recent decades. The incidence of EAC has also increased significantly globally. The endoscopic surveillance intervals as set out by international guidelines are helpful in stratifying the risk of BE and treating its associated dysplasia. In the future, prognostic biomarkers can serve as novel tools for detecting the progression of BE. The epidemiology and surveillance strategy of BE in the Asian region likely contrast those in the Western countries, and further research within the Asian populations will help to better diagnose and treat patients of BE.

## Declaration of conflict of interest

The authors declare no conflict of interest.
